# ct-validation: an open framework for benchmarking target-indication evidence against clinical trial outcomes

**DOI:** 10.1093/bioinformatics/btag788

**Published:** 2026-10-20

**Authors:** Klim Kostiuk, Daniel Igumnov, Peter Fedichev, Amir Feizi

**Affiliations:** Translational AI and Discovery, GERO PTE. LTD., 133 Cecil Street 14-01 Keck Seng Tower, 069535, Singapore; Translational AI and Discovery, GERO PTE. LTD., 133 Cecil Street 14-01 Keck Seng Tower, 069535, Singapore; Translational AI and Discovery, GERO PTE. LTD., 133 Cecil Street 14-01 Keck Seng Tower, 069535, Singapore; Translational AI and Discovery, GERO PTE. LTD., 133 Cecil Street 14-01 Keck Seng Tower, 069535, Singapore

## Abstract

**Summary:**

Human genetic evidence provides a strong foundation for selecting drug targets. However, consistent benchmarking of target-indication evidence against clinical trial outcomes has been hindered by proprietary data and customized pipelines. To address this, we present the Python package ct-validation, which evaluates whether target-indication pairs supported by specific evidence sources are enriched for clinical progression. This package computes relative success (RS) and odds ratios with confidence intervals across clinical phase transitions. It also supports ontology-based semantic disease matching and offers interfaces in Python, on the command line, and via the Model Context Protocol. By remapping the benchmark from Minikel *et al.* (2024) to the Experimental Factor Ontology, we reproduced their per-transition enrichment, with a maximum difference of only 1.4% across any transition. In a systematic 3 × 3 benchmark that independently varies the genetic evidence and clinical trial sources, we found that a single curated open clinical trial source reproduces the signal of the proprietary comparator (RS = 2.92 versus 2.65; 2.34 versus 2.55 without oncology). However, aggregating open clinical trial registries compresses this signal (RS = 1.52); this loss is primarily driven by the breadth of the indication data rather than the genetic evidence.

**Availability and implementation:**

ct-validation is implemented in Python (version 3.11 or higher), distributed via pip, and freely available at https://github.com/gero-science/ct-validation, with support for Python, command-line, and Model Context Protocol interfaces. Archived code: https://doi.org/10.5281/zenodo.22901497. Benchmark data: https://doi.org/10.5281/zenodo.22901451

## 1 Introduction

Most drug development programmes fail before approval, making target selection central to therapeutic discovery. Human genetics is one of the strongest signals for linking genes to disease and for target prioritization (Plenge *et al.* 2013, Sadler *et al.* 2023), and drug mechanisms with genetic support are more likely to succeed (Nelson *et al.* 2015, King *et al.* 2019); Minikel *et al.* (2024) recently estimated that genetically supported target-indication pairs are ∼2.6 times more likely to progress from phase I to approval. That estimate is hard to reproduce or extend because it depends on proprietary data and bespoke preprocessing, and existing implementations are embedded in one-off analyses. Concurrent frameworks such as TargetBench (Leung *et al.* 2026) score predicted target lists based on their recovery of established targets and several measures of development potential, including druggability, but do not test enrichment for phase-transition success in clinical trials or provide a reusable, open implementation.

We present ct-validation, a Python package that implements phase-transition enrichment and semantic disease matching in reusable form, with parsers for major public genetic evidence and clinical trial databases harmonized through the Experimental Factor Ontology (EFO). The genetic evidence and the clinical trial data can be provided independently, allowing the user to isolate what each contributes to the observed signal. We demonstrate it by (i) replicating the Minikel *et al.* (2024) benchmark on their data remapped from MeSH to EFO and (ii) a 3×3 design that varies each axis across the published comparator, a single curated open source, and an aggregate of open sources, localizing the loss of enrichment in an open-data setting.

## 2 Methods

### 2.1 Overview of the framework

ct-validation tests whether target-indication pairs supported by a given evidence source are enriched for successful clinical progression. A drug’s *indication* is the disease it is developed to treat, so a clinical target-indication pair and a target-disease evidence pair describe the same gene-disease relationship from the clinical and evidence sides, respectively. Inputs are (i) clinical target-indication pairs annotated with the highest phase reached, optionally, whether that phase is still ongoing; (ii) target-disease evidence pairs; and (iii) an optional disease-similarity lookup that reconciles the two; outputs are enrichment statistics [relative success (RS) and odds ratios (OR) with confidence intervals (CIs)] per phase transition and for phase I to approval.

### 2.2 Data source parsers

Reproducible scripts parse public databases into standardized target-disease evidence tables using EFO as the key identifier and its imported ontologies (EFO 3.84.0); MeSH-coded indications are remapped through the OxO crosswalk, and identifiers already in the namespace are kept as recorded rather than rewritten. Genetic evidence combines four sources: GWAS Catalog (Cerezo *et al.* 2025) at genome-wide significance ($P<5\times 10^{-8}$); ClinVar (Landrum *et al.* 2014) pathogenic/likely-pathogenic variants at high review confidence (somatic, susceptibility, and response entries excluded); Open Targets Platform 25.12 (Ochoa *et al.* 2023) from association_by_datasource_direct (direct rather than ontology-propagated scores; maximum across genetic datasources, ≥0.5); and Genebass (Karczewski *et al.* 2022) at $P<10^{-7}$. Direct scores are used because ontology propagation and similarity matching (Section 2.3) address the same granularity mismatch, so combining them would double-count evidence. The nine germline data sources are listed in the Methods, available as supplementary data at *Bioinformatics* online. Somatic data sources (eva_somatic, intogen) are excluded from the headline, matching the germline-only convention of Minikel *et al.* (2024), and re-enabled with a flag (Table 6, available as supplementary data at *Bioinformatics* online). Oncology indications are retained, as in Minikel *et al.* (2024). In the stratified analysis (Table 4 and Fig. 7, available as supplementary data at *Bioinformatics* online), oncology means MeSH tree C04, their definition, or the neoplasm branch of EFO (EFO: 0000616), which covers the tables without MeSH annotation.

Clinical trial data combines target-drug links [ChEMBL, Open Targets known_drug, STITCH (Szklarczyk *et al.* 2016), DGIdb (Cannon *et al.* 2024), TrialPanorama (Wang *et al.* 2025); ChEMBL/TrialPanorama at pChEMBL≥7.0, STITCH high-confidence links] with drug-indication links carrying maximum phase (Open Targets and TrialPanorama, interventional studies only; ChEMBL excluded here as its indications carry no trial status). As a proprietary comparator, we use the Citeline (Pharmaprojects) table from Minikel *et al.* (2024) (CC BY 4.0, with Citeline’s permission). Their genetic-insight filter on indications is applied to this arm; the open trial tables have no such annotation and are used in full. Drug identifiers are harmonized by Union–Find, the two legs joined to yield target-indication pairs with maximum phase, and analyses restricted to protein-coding genes from the Open Targets 25.12 target table (20 097 symbols). Per-source composition and overlaps are in Figs 1 and 2 and Table 2, available as supplementary data at *Bioinformatics* online.

**Figure 1:**
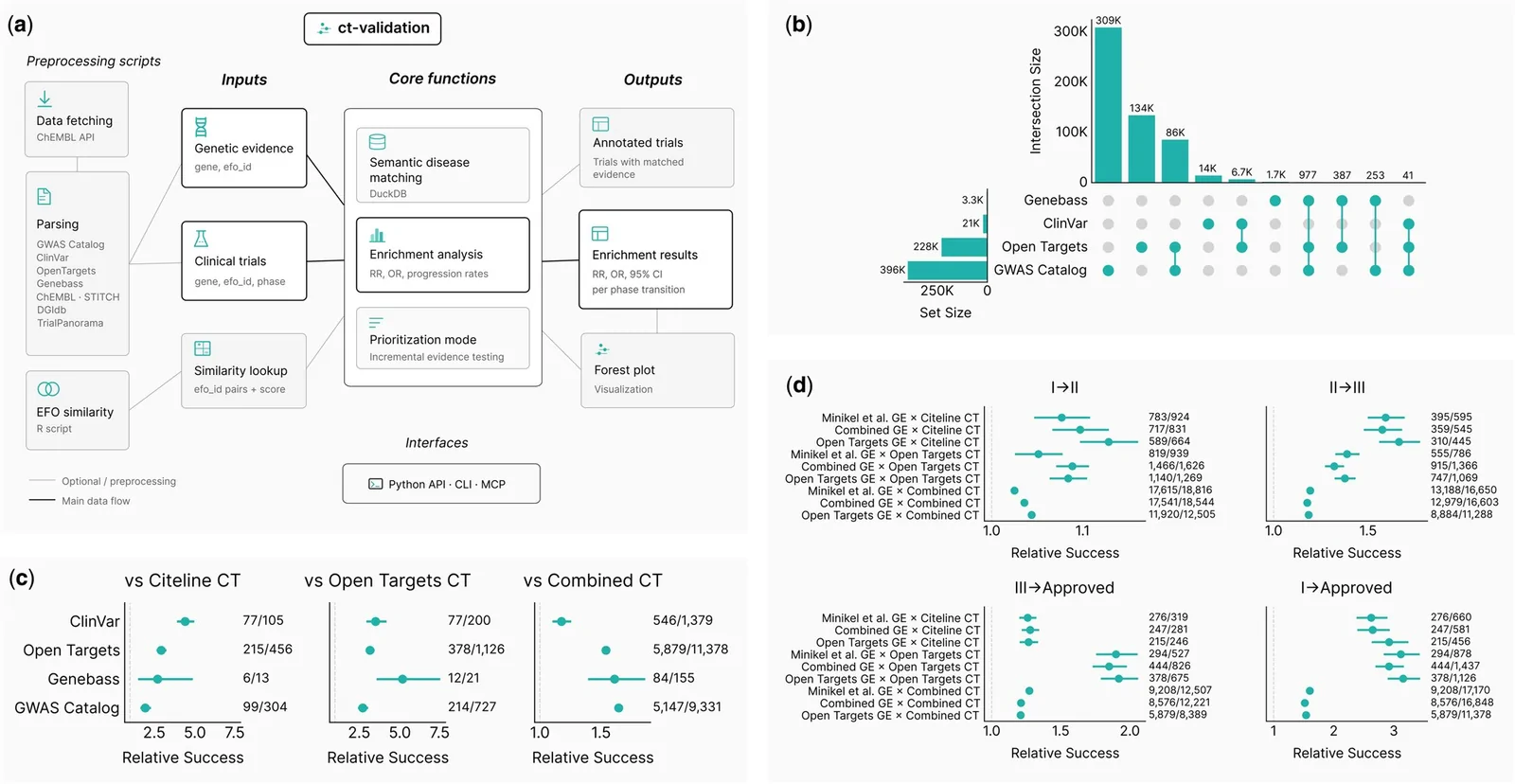
Overview of the ct-validation framework and benchmark results. (a) Workflow for assembling harmonized inputs and computing enrichment statistics, including ontology-based disease matching and RS/OR estimation. (b) Overlap among genetic evidence sources used in the open benchmark; horizontal bars give the number of target-disease pairs each source contributes, and the dotted columns mark set intersections. (c) Source-specific phase I to approval RS for each genetic evidence source against all three clinical trial arms (Citeline, Open Targets known_drug, and the combined open table). (d) The 3×3 benchmark: genetic evidence and clinical trial sources each varied across the published comparator, a single curated open source, and the aggregate of open sources, with point estimates and 95% CI; per-cell contingency counts are given in Table 1, available as supplementary data at *Bioinformatics* online. In (c) and (d), the fraction beside each estimate is $x_{\text{yes}}/n_{\text{yes}}$, the supported pairs reaching the target phase over those entering the transition. ‘Combined CT’ denotes the aggregated open clinical trial table built from Open Targets known_drug, TrialPanorama, ChEMBL, DGIdb, and STITCH (2 236 441 target-indication pairs, 5336 genes, 4568 indications before the protein-coding gene universe filter).

### 2.3 Semantic disease matching

We precompute pairwise EFO similarity from ontology structure, averaging normalized Resnik (Resnik 1999) and Lin (Lin 1998) information-content measures [via the ontologySimilarity R package (Greene *et al.* 2017); self-similarity 1]. A *storage* threshold of 0.5 governs which pairs are retained in the lookup; a *matching* threshold of 0.8, the package default, defines genetic support and is used throughout. A clinical pair (gene, disease) is supported when the evidence contains a pair (gene, disease${\;}^{\prime }$) whose disease scores are at or above the matching threshold (exact matches, scoring 1, included); without a lookup, support is exact gene-disease matching. A sweep over 0.5–1.0 shows that raising the matching threshold increases the estimate and reduces the supported count (Table 3 and Fig. 6, available as supplementary data at *Bioinformatics* online).

### 2.4 Enrichment analysis

For each transition (I→II, II→III, III→Approved, I→Approved), pairs that reach at least the initial phase are divided into supported and unsupported groups. Programmes that have not yet been determined are censored for each transition; specifically, any pair still active below the target phase is excluded from the analysis rather than counted as a failure, as in Minikel *et al.* (2024).

In each group, if *x* out of *n* pairs advance, the risk ratio (RR) is calculated as $\text{RR}=\frac{x_{\text{yes}}/n_{\text{yes}}}{x_{\text{no}}/n_{\text{no}}}$. This ratio serves as the RS metric of Nelson *et al.* (2015) and Minikel *et al.* (2024), and we will refer to it as RS throughout this document (it is exposed in the package as rr).

We report Katz log 95% CIs for RS and the OR, calculated as $\text{OR}=\frac{x_{\text{yes}}(n_{\text{no}}-x_{\text{no}})}{x_{\text{no}}(n_{\text{yes}}-x_{\text{yes}})}$. For the OR, we apply Woolf logit CIs and a Haldane–Anscombe correction for zero cells, along with a secondary gene-clustered bootstrap approach (see Tables 1 and 10, available as supplementary data at *Bioinformatics* online).

### 2.5 Software implementation

ct-validation exposes validate() as a single entry point, a command-line interface, and Model Context Protocol (MCP) server with two tools: ct_validate (clinical trial pairs, genetic evidence pairs and an optional similarity lookup, as Parquet paths or inline records, returning per-transition counts, rates, RR and OR with 95% CIs and a Fisher *P*-value) and expand_disease_set (expands EFO identifiers to their semantic neighbourhood). It supports Parquet I/O, batched comparison of evidence sources, and forest plot visualization. An optional baseline_evidence mode quantifies the incremental value of a new source over an existing baseline, excluding baseline-only pairs. The workflow and architecture are shown in Fig. 1.

## 3 Results

### 3.1 Replication of the published enrichment benchmark

We investigated whether the enrichment reported by Minikel *et al.* (2024) is captured by an independent EFO-based implementation. Utilizing their genetic evidence and data from Citeline (Pharmaprojects), which we remapped from MeSH to EFO, we matched their cohort by applying censoring. Our findings revealed a comparable, though not identical, signal. The discrepancies arise from differences in the ontologies used, disease matching, and curation processes; for instance, our methodology incorporates ClinVar, excludes PICCOLO, and employs automated rather than manual curation.

When examining the per-transition estimates, the agreement is nearly exact: for the transition from I-to-II, we found an enrichment of 1.08 [1.05, 1.11], compared to their value of 1.08; for II-to-III, it was 1.59 [1.50, 1.69] versus 1.57 (a 1.4% difference); and from III to Approved, the values were 1.26 [1.20, 1.32] compared to 1.28 (a 1.2% difference). Their published per-transition estimates use the same censoring convention, making these three transitions directly comparable. The composite metric of phase I to approval is sensitive to the censoring convention used. Minikel *et al.* (2024) applied censoring once at phase I entry, while we implemented it at each transition. When matched to their censoring convention, our metric yields an enrichment of 2.72 [2.45, 3.03], compared to their 2.63 [2.31, 3.00], resulting in a 3.4% difference. In our analysis, using our own per-transition censoring method, we found an enrichment of 2.63 [2.38, 2.89] (see Fig. 1d), while the uncensored value was 2.82 [2.53, 3.14]. All four estimates overlap as shown in Tables 1 and 8, available as supplementary data at *Bioinformatics* online.

### 3.2 Differential impact of genetic and clinical data on enrichment

To separate the contributions of each data axis, we independently substituted the genetic evidence and clinical trial sources in a systematic 3×3 design. Each axis had three levels: the Minikel *et al.* (2024) comparator, a single curated open-source dataset (Open Targets), and our aggregate of open-source datasets (GWAS Catalog, Open Targets, ClinVar, and Genebass).

Substituting the published genetic evidence with the combined open set, while keeping the clinical data from Citeline, resulted in an essentially unchanged phase I-to-approval signal (RS = 2.65 [2.39, 2.93]), compared with the 2.63 [2.38, 2.89] replication. However, when the clinical data were substituted instead, the results were compressed: with genetic evidence held at Minikel’s, the combined open clinical trial table (Combined CT) yielded an RS of 1.60 [1.58, 1.63], and the fully open configuration produced an RS of 1.52 [1.50, 1.54] (Fig. 1d). The clinical trial axis dominates: substituting the genetic evidence moves the phase I-to-approval estimate by 5%–11% depending on the trial table, substituting the trial table by 92%–105% (Table 1, available as supplementary data at *Bioinformatics* online).

This compression is not a result of using the open dataset itself. A single curated open source is comparable to the proprietary comparator: using the same genetic evidence, Open Targets known_drug yields an RS of 2.92 [2.69, 3.17], compared to Citeline’s 2.65 [2.39, 2.93]; without oncology indications they give 2.34 [2.15, 2.55] (known_drug) and 2.55 [2.29, 2.85] (Citeline), while the aggregated table falls to 1.31 [1.29, 1.33] (Table 4, available as supplementary data at *Bioinformatics* online). When varying one clinical leg at a time, the indication leg primarily influences the estimate, while the target-drug leg does not. Open Targets indications provide an RS of 2.09 [2.02, 2.16] versus TrialPanorama’s 1.55 [1.53, 1.57] (with disjoint intervals). The aggregate result aligns with TrialPanorama’s estimate of 1.52, whereas the five target-drug sources span only from 1.47 to 1.70 (Table 7 and Fig. 3, available as supplementary data at *Bioinformatics* online).

Part of this outcome is mechanical, as a broader indication set raises the base rate (from 0.14 to 0.36), thereby compressing the RR by construction. However, this effect is specific to the indication census; the OR, which is not affected by a rising base rate in the same manner, also decreases (from 2.53 to 2.25). Therefore, we frame the result as curated versus aggregated rather than open versus proprietary.

### 3.3 Variation in enrichment across genetic evidence sources

We next examined the enrichment of individual genetic evidence sources (Fig. 1c, Table 11, available as supplementary data at *Bioinformatics* online). Compared with Citeline, the Mendelian evidence was the strongest. ClinVar showed the highest ratio of phase I-to-approval (RS) at 4.39 [3.89, 4.96], followed by Open Targets at 2.92 [2.63, 3.24] and the GWAS Catalog at 1.94 [1.64, 2.29]. These intervals do not overlap. Genebass, which is based on only 13 pairs, had an RS of 2.70 [1.50, 4.86]. This ranking is consistent with previous reports that indicate Mendelian signals have stronger predictive value than common-variant signals (Nelson *et al.* 2015, Minikel *et al.* 2024), likely due to higher-penetrance gene-disease relationships and more direct causal mechanisms.

When we compared the combined open clinical trial table, all sources remained enriched; however, the effect sizes were compressed and the order inverted. The GWAS Catalog for common variants now showed the strongest signal with an RS of 1.64 [1.61, 1.67], followed by Open Targets at 1.54 [1.51, 1.57] and ClinVar, which had the most attenuation at 1.18 [1.10, 1.26]. Genebass had an RS of 1.61 [1.39, 1.86], overlapping with the GWAS Catalog and Open Targets but not with ClinVar. Within Open Targets, increasing the association score threshold slightly improved the RS, albeit at the expense of coverage (Figs 4 and 5, available as supplementary data at *Bioinformatics* online).

A recent large-scale study from Open Targets (Tsepilov *et al.* 2026) ran a similar analysis in which the clinical trial side is built from ChEMBL-derived target-indication pairs, and reported different point estimates. Their genetic support is also constructed differently from ours: locus-to-gene (L2G) assignment over GWAS credible sets rather than a threshold on the released association score, on Platform 25.06 rather than 25.12. Reproducing their cohort definition as a convergence check (Table 5 and Fig. 8, available as supplementary data at *Bioinformatics* online), our three genetic evidence definitions yielded RS values between 2.57 and 2.94, bracketing their reported 2.76 [2.48, 3.08]; our combined set is within 2.3% and every interval overlaps with theirs. Thus, two independent constructions of genetic support converge on the same effect.

## 4 Discussion

The ct-validation framework offers a reusable, modular approach to evaluating whether target-indication evidence correlates with clinical success. This allows for consistent comparisons across rapidly evolving evidence sources and prioritization methods. The framework is flexible, accommodating various types of evidence, including genetic data, omics-derived information, computational findings, and data curated from resources such as ChEMBL. The baseline_evidence mode assesses the additional value of a new source compared to an existing baseline, while the MCP interface facilitates agent-based discovery workflows.

Our replication successfully retrieves the benchmark set by Minikel *et al.* (2024) for each transition. The 3×3 benchmark indicates that the clinical trial information, rather than the assembly of genetic evidence, primarily drives the compression of open data.

Several limitations should be acknowledged. Estimates rely on disease harmonization, gene mapping, and trial annotations. A key limitation of the aggregated benchmark lies in the indication leg; pooling registry indications sacrifices precision for broader coverage. Additionally, direction-of-effect information is not consistently available across public resources and is often ignored. For instance, Genebass reports signed burden effect sizes (BETA_Burden), which provide the direction of the effect, but our parser discards these values. On the positive side, the open benchmark encompasses a much larger number of genes and indications (see Table 9, available as supplementary data at *Bioinformatics* online). As noted in previous studies, enrichment should be considered a relative measure of target success rather than an absolute one.

## Supplementary Material

btag788_Supplementary_Data

## Data Availability

The ct-validation package and related materials are available at https://github.com/gero-science/ct-validation. An archived version is available at https://doi.org/10.5281/zenodo.22901497. To reproduce the benchmarks, download the parsed datasets at https://doi.org/10.5281/zenodo.22901451. Extracting the archive will allow you to recreate all figures and tables from notebooks/benchmark.py. Details on licenses and download instructions are in DATA_SOURCES.md, and all resources are publicly accessible.
