# Supplementary material for: ct-validation: an open framework for benchmarking target-indication evidence against clinical trial outcomes
Source: Bioinformatics. 2026 Oct 20;42(10):btag788. doi: 10.1093/bioinformatics/btag788 (PMC13719226; doi:10.1093/bioinformatics/btag788)
Supplement: Supplementary file 1 [file btag788_supplementary_data.pdf]

Supplementary Data

`ct-validation`: an open framework for benchmarking gene–indication evidence against clinical trial outcomes

Klim Kostiuk Daniel Igumnov Peter Fedichev Amir Feizi

Notation

Genetic-evidence arms. *Minikel et al. GE* is the genetic evidence released with Minikel et al. (2024), remapped to EFO. *Open Targets GE* is Open Targets Platform 25.12 `association_by_datasource_direct`, taking the maximum score across the nine germline genetic datasources at ≥ 0.5. *Combined GE* is the union of GWAS Catalog, ClinVar, Open Targets and Genebass.

Clinical-trial arms. *Citeline CT* is Citeline (Pharmaprojects), remapped from MeSH to EFO. *Open Targets CT* is the Open Targets `known_drug` table. *Combined CT* is the aggregated open table built from Open Targets `known_drug`, TrialPanorama, ChEMBL, DGIdb and STITCH. An arm written *A GE* $\times$ *B CT* tests evidence arm A against trial arm B.

Legs. The target–drug leg and the drug–indication leg are the two joins that build a target–indication pair. Generated figure panels and table cells carry the pipeline's own column names, so `gene_drug` and `gene-drug` there denote the target–drug leg.

Counts. For a transition out of phase ϕ, n_{yes} is the number of target–indication pairs with genetic evidence that reached phase ϕ and are not censored, and x_{yes} the subset of those that reached the later phase; n_{no} and x_{no} are the same counts for pairs without genetic evidence. *Max* ϕ is the highest phase a pair reached.

Censoring. *cens.* is per-transition censoring: a pair still running below the target phase is dropped from that transition, its outcome being undetermined. *uncens.* drops nothing, so a still-running pair counts as a failure. Table S8 adds a third convention, entry-gate censoring, which censors only at phase I entry.

Supplementary Methods

Genetic-evidence datasources. For Open Targets, we take the maximum score across nine germline datasources at ≥ 0.5: `gwas_credible_sets`, `eva`, `gene_burden`, `genomics_england`, `gene2phenotype`, `clingen`, `uniprot_variants`, `uniprot_literature` and `orphanet`. The two somatic datasources excluded from the headline (available behind the `--include-somatic` flag) are `eva_somatic` and `intogen`.

Statistical definitions. Relative success (RS) is the risk ratio of phase advancement, with a 95% confidence interval by the Katz log method:

$$RS = \frac{x_{\text{yes}}/n_{\text{yes}}}{x_{\text{no}}/n_{\text{no}}}, \qquad \text{CI}_{RS} = \exp\!\left(\ln RS \pm z_{0.975}\sqrt{\frac{1}{x_{\text{yes}}} - \frac{1}{n_{\text{yes}}} + \frac{1}{x_{\text{no}}} - \frac{1}{n_{\text{no}}}}\right).$$

The odds ratio and its Woolf logit interval are

$$\text{OR} = \frac{x_{\text{yes}}(n_{\text{no}} - x_{\text{no}})}{x_{\text{no}}(n_{\text{yes}} - x_{\text{yes}})}, \qquad \text{CI}_{OR} = \exp\!\left(\ln \text{OR} \pm z_{0.975}\sqrt{\frac{1}{x_{\text{yes}}} + \frac{1}{n_{\text{yes}}-x_{\text{yes}}} + \frac{1}{x_{\text{no}}} + \frac{1}{n_{\text{no}}-x_{\text{no}}}}\right).$$

When any contingency-table cell is zero, a Haldane–Anscombe correction of 0.5 is applied to all four cells before estimation.

Supplementary Figures

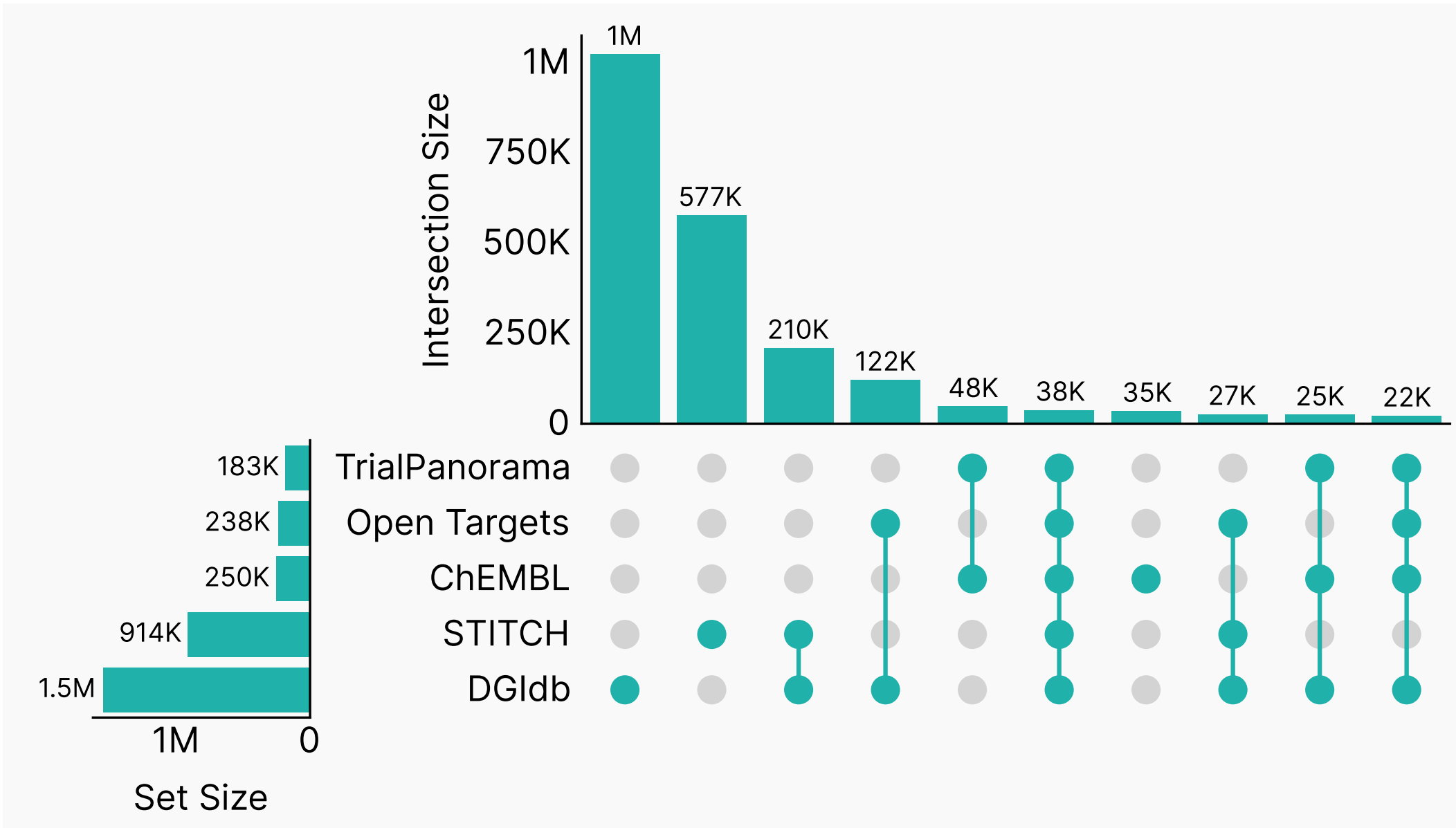

Figure S1: Overlap of target–drug sources across target–indication pairs. UpSet plot of the target–indication pairs contributed by DGIdb, STITCH, Open Targets `known_drug`, ChEMBL and TrialPanorama on the target–drug leg; bar heights are intersection sizes, and set sizes are computed over every combination before truncation. Only the ten largest of 31 intersections are drawn, hiding 107,301 of 2,236,441 pairs (4.8%). *Alt-text:* an UpSet bar-and-dot plot of the overlap of target–indication pairs among five target–drug sources.

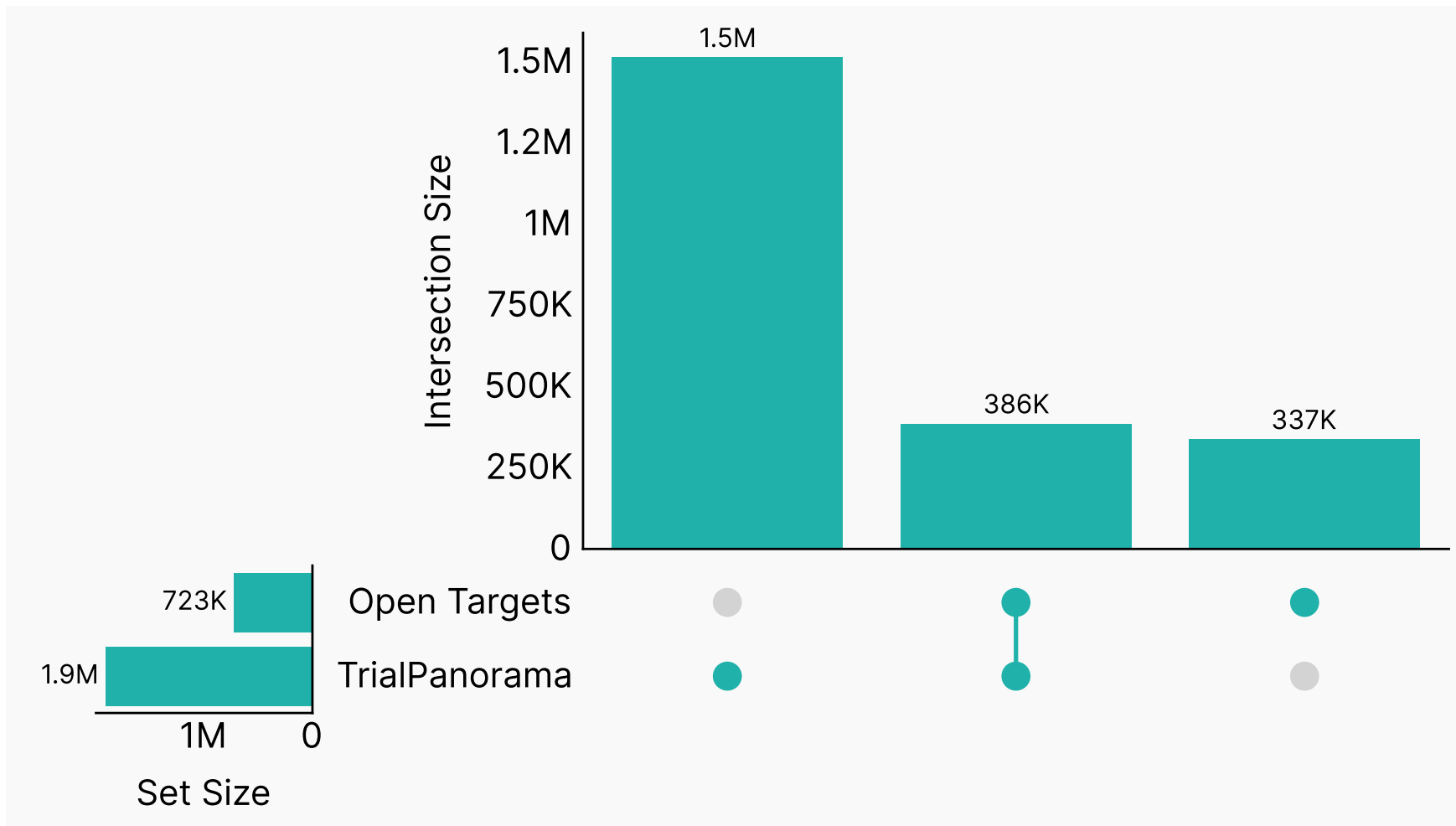

Figure S2: Overlap of the two drug–indication sources. UpSet plot of the target–indication pairs contributed by TrialPanorama and Open Targets on the drug–indication leg. ChEMBL is excluded from the aggregated indication leg because its indications carry no trial status to censor on (Methods 2.2 of the main text). *Alt-text:* an UpSet bar-and-dot plot of the overlap of target–indication pairs between two drug–indication sources.

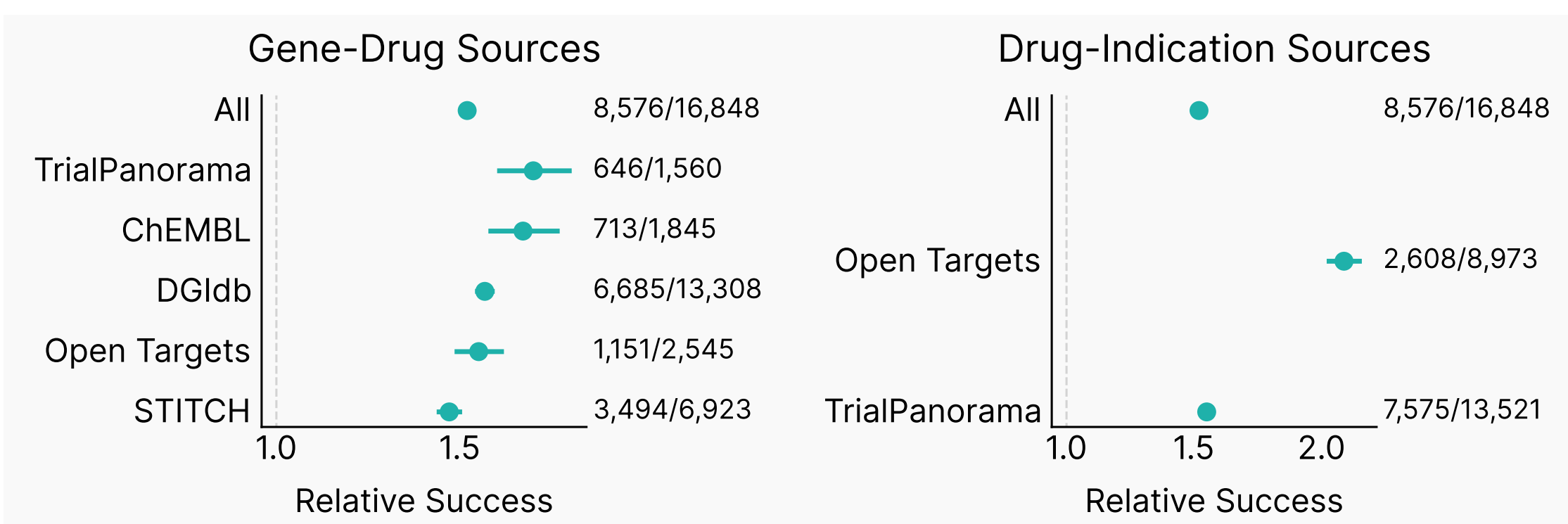

Figure S3: Enrichment across individual clinical-trial sources. Phase I to approval relative success on combined genetic evidence, split by leg: target–drug sources (left) and drug–indication sources (right). Points are RS, bars 95% Katz CI. The fraction beside each estimate is $x_{\text{yes}}/n_{\text{yes}}$, as in Figure 1, and has the same meaning in every forest plot in this supplement. The indication leg decides the estimate; the target–drug leg does not. Corresponds to Supplementary Table S7. *Alt-text:* a forest plot of phase I to approval relative success for each clinical-trial source, grouped by leg.

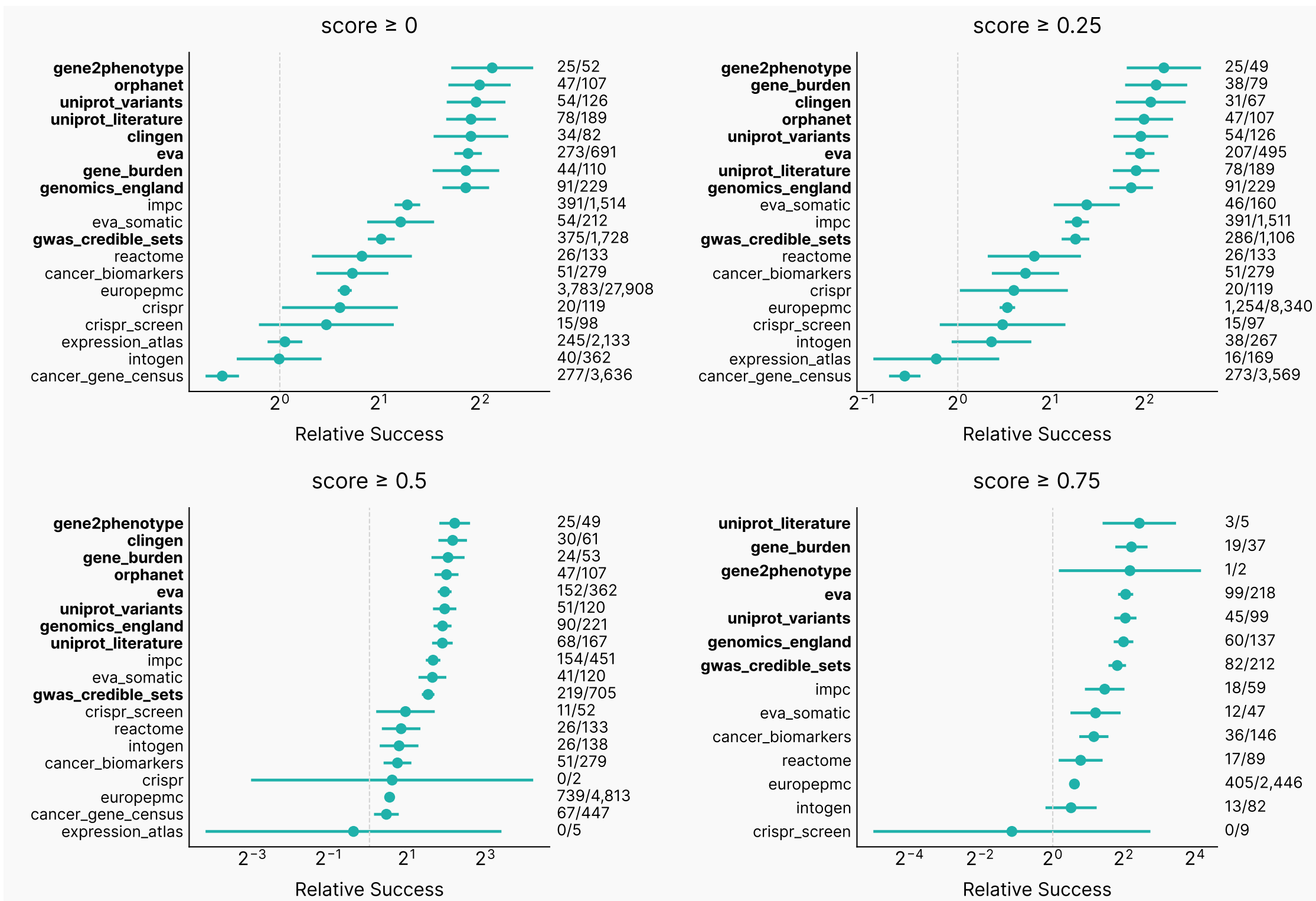

Figure S4: Enrichment across Open Targets datasources. Phase I to approval relative success for each Open Targets datasource at a range of association-score thresholds. The panels span the datasources the Platform publishes at each cutoff, not only the genetic ones, so the higher-threshold panels carry fewer rows; bold marks the nine germline datasources combined in the main analysis. `chembl` and the `overall` aggregate are omitted because they carry approved pairs by construction, which empties the unsupported arm at the lower cutoffs and leaves the estimate undefined there; both remain in `ot_subsource_comparison.csv`. The eight largest estimates at score ≥ 0 are all germline, from `gene2phenotype` (4.35) to `genomics_england` (3.62), the ninth being `impc` (2.42). *Alt-text:* a forest plot of relative success for individual Open Targets datasources across four score thresholds.

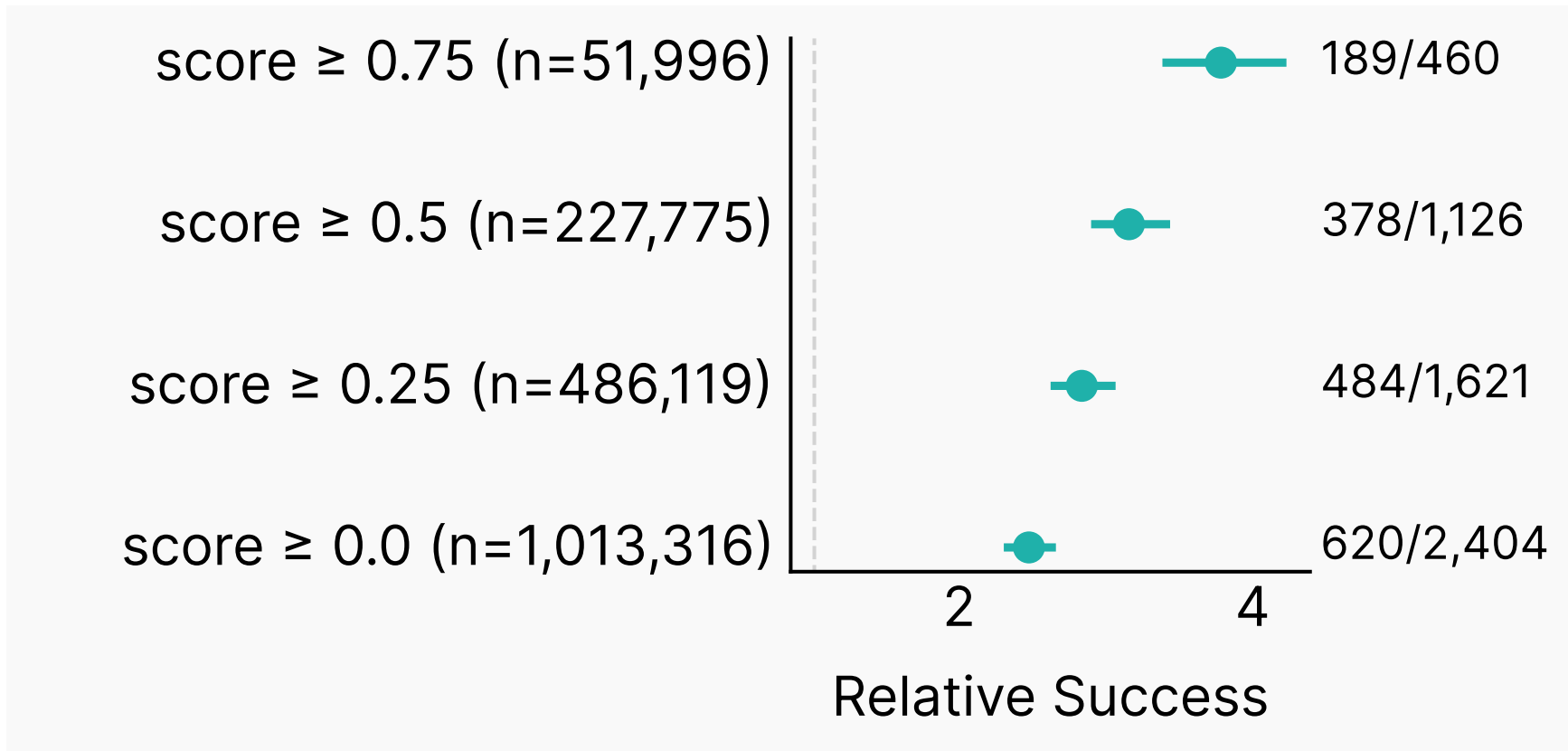

Figure S5: Effect of the Open Targets association-score threshold on enrichment. Phase I to approval relative success at each association-score cutoff, computed over the nine germline genetic-evidence datasources used throughout (Supplementary Methods), not the full set of Platform datasources broken down in Fig. S4. The n in each tick label is the number of genetic-evidence pairs surviving that cutoff. Raising the cutoff raises RS and shrinks both n and n_{yes}. *Alt-text:* a forest plot of relative success at four Open Targets association-score thresholds, with pair counts falling as the threshold rises.

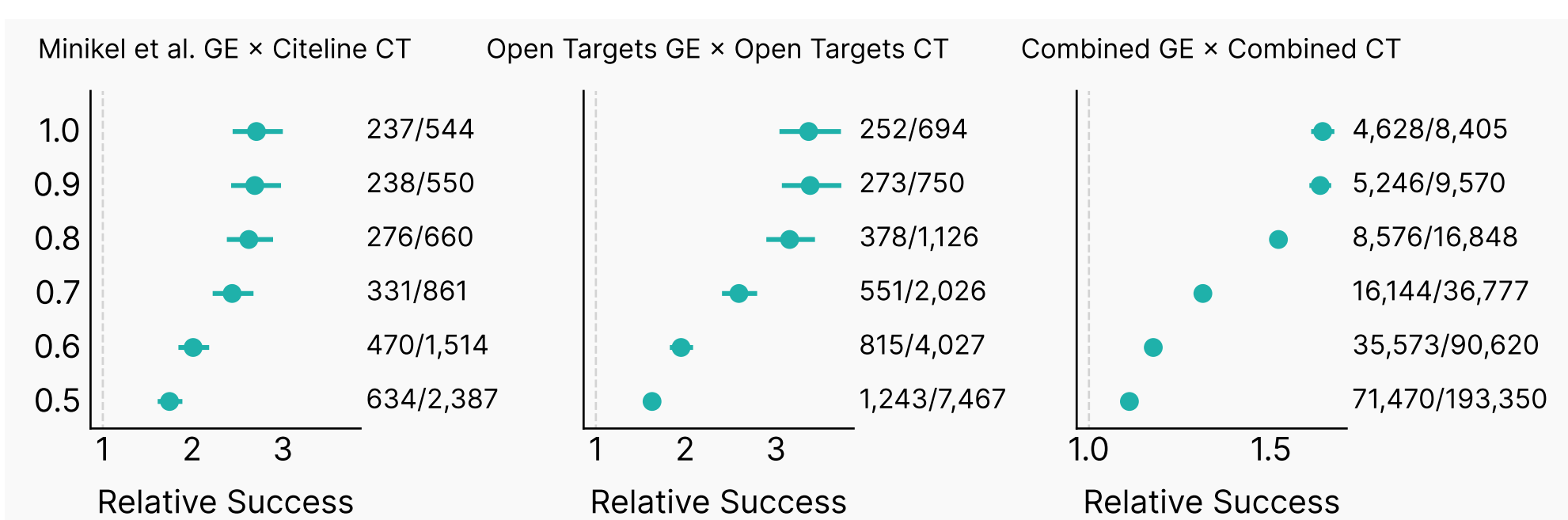

Figure S6: Effect of the semantic-similarity matching threshold on enrichment. Phase I to approval relative success for three benchmark arms as the matching threshold on the vertical axis is swept from 0.5 to 1.0; 0.8 is the package default. Corresponds to Supplementary Table S3. *Alt-text:* a forest plot of relative success against the semantic-similarity matching threshold for three benchmark arms.

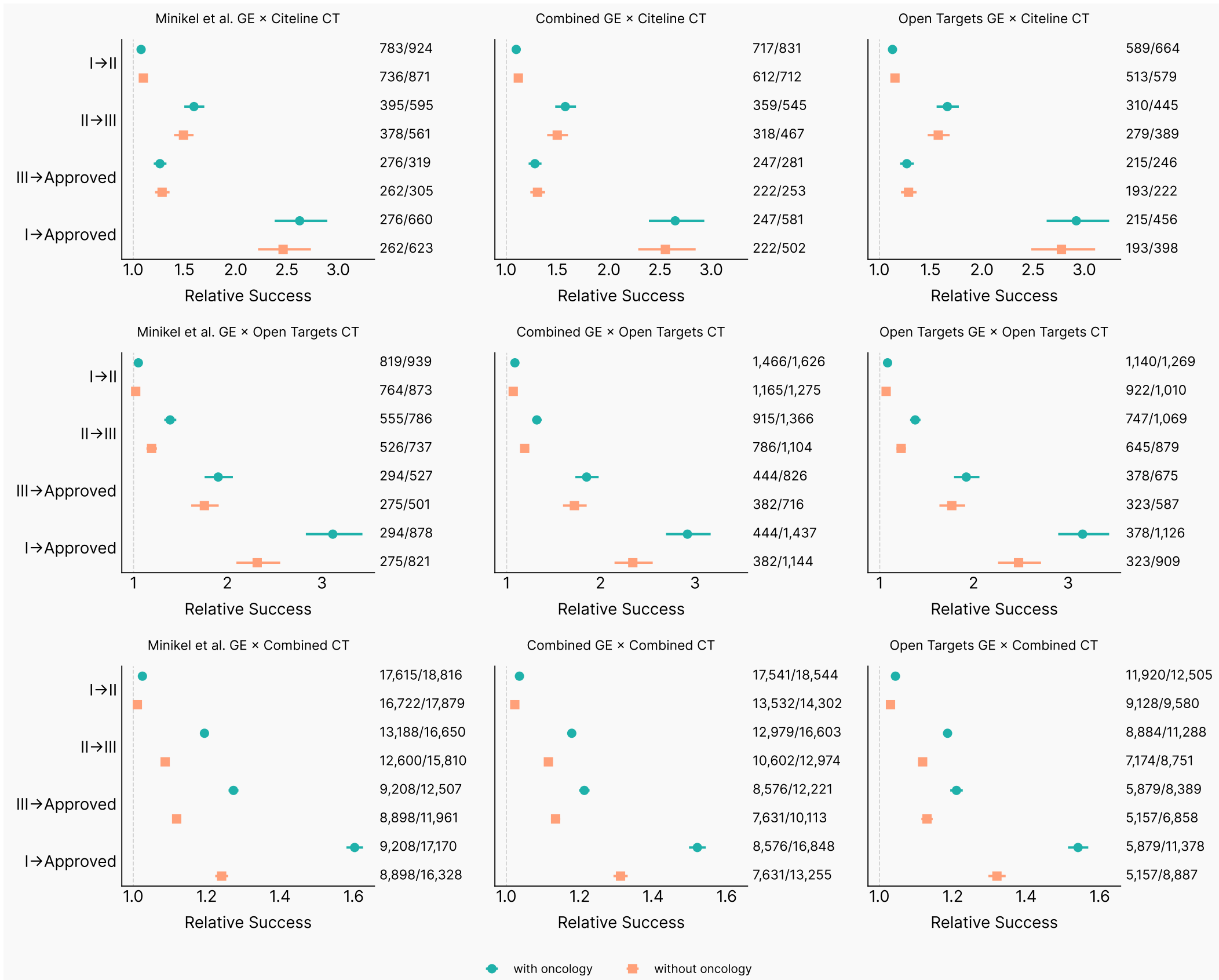

Figure S7: Oncology-stratified enrichment across the 3×3 design. Relative success with (circles) and without (squares) oncology indications at all four transitions; the x scale differs between rows. Supplementary Table S4 tabulates the phase I to approval row. *Alt-text:* a 3 × 3 grid of forest plots comparing relative success with and without oncology across phase transitions.

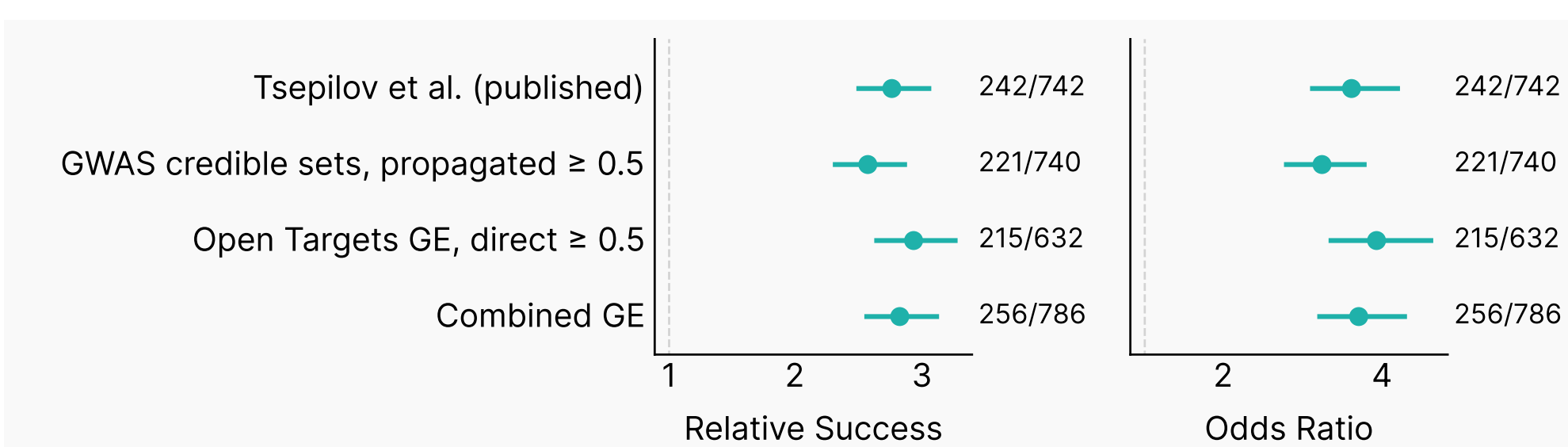

Figure S8: Convergence with Tsepilov et al. (2026). Phase I to approval under their cohort definition for three genetic-evidence definitions, against their published value; the left panel is on the relative-success scale and the right on the odds-ratio scale, each identified by its axis label. Corresponds to Supplementary Table S5. *Alt-text:* two forest plots comparing three genetic-evidence definitions with the Tsepilov et al. published estimate, one on the relative-success scale and one on the odds-ratio scale.

Supplementary Tables

All tables use the notation defined above, and per-transition censoring unless stated otherwise. Each is generated from a corresponding CSV in `notebooks/output/` of the code deposit, where the same quantities are available at full precision; percentages quoted in the main text are computed from those unrounded values rather than from the two-decimal entries printed here.

Table S1: Full 3×3 benchmark. Every genetic-evidence $\times$ clinical-trial combination at each phase transition, under both censoring conventions, with relative success (RS), its Katz 95% CI and clustered-bootstrap 95% CI (10,000 replicates, seed 0, genes resampled), and the odds ratio with its Woolf 95% CI.

Experiment	Cens.	Trans.	$x_{\text{yes}}/n_{\text{yes}}$	$x_{\text{no}}/n_{\text{no}}$	RS	RS 95% CI (Katz)	RS 95% CI (boot)	OR	OR 95% CI
Combined GE × Citeline CT	cens.	I→II	717/831	15,820/20,103	1.10	[1.07, 1.13]	[1.05, 1.14]	1.70	[1.39, 2.08]
Combined GE × Citeline CT	cens.	II→III	359/545	4,559/10,903	1.58	[1.48, 1.68]	[1.44, 1.72]	2.69	[2.24, 3.22]
Combined GE × Citeline CT	cens.	III→Approved	247/281	2,225/3,237	1.28	[1.22, 1.34]	[1.20, 1.37]	3.30	[2.29, 4.77]
Combined GE × Citeline CT	cens.	I→Approved	247/581	2,225/13,864	2.65	[2.39, 2.93]	[2.29, 3.06]	3.87	[3.26, 4.59]
Combined GE × Citeline CT	uncens.	I→II	717/927	15,820/23,248	1.14	[1.10, 1.18]	[1.09, 1.19]	1.60	[1.37, 1.87]
Combined GE × Citeline CT	uncens.	II→III	359/717	4,559/15,820	1.74	[1.61, 1.88]	[1.55, 1.92]	2.48	[2.13, 2.88]
Combined GE × Citeline CT	uncens.	III→Approved	247/359	2,225/4,559	1.41	[1.31, 1.52]	[1.26, 1.56]	2.31	[1.84, 2.91]
Combined GE × Citeline CT	uncens.	I→Approved	247/927	2,225/23,248	2.78	[2.48, 3.12]	[2.35, 3.25]	3.43	[2.95, 4.00]
Combined GE × Combined CT	cens.	I→II	17,541/18,544	1,934,679/2,118,134	1.04	[1.03, 1.04]	[1.03, 1.04]	1.66	[1.56, 1.77]
Combined GE × Combined CT	cens.	II→III	12,979/16,603	1,184,685/1,785,165	1.18	[1.17, 1.19]	[1.16, 1.19]	1.82	[1.75, 1.88]
Combined GE × Combined CT	cens.	III→Approved	8,576/12,221	623,052/1,076,226	1.21	[1.20, 1.23]	[1.19, 1.24]	1.71	[1.65, 1.78]
Combined GE × Combined CT	cens.	I→Approved	8,576/16,848	623,052/1,860,161	1.52	[1.50, 1.54]	[1.47, 1.57]	2.06	[2.00, 2.12]
Combined GE × Combined CT	uncens.	I→II	17,541/18,787	1,934,679/2,164,565	1.04	[1.04, 1.05]	[1.04, 1.05]	1.67	[1.58, 1.77]
Combined GE × Combined CT	uncens.	II→III	12,979/17,541	1,184,685/1,934,679	1.21	[1.20, 1.22]	[1.19, 1.23]	1.80	[1.74, 1.86]
Combined GE × Combined CT	uncens.	III→Approved	8,576/12,979	623,052/1,184,685	1.26	[1.24, 1.27]	[1.23, 1.28]	1.76	[1.69, 1.82]
Combined GE × Combined CT	uncens.	I→Approved	8,576/18,787	623,052/2,164,565	1.59	[1.56, 1.61]	[1.53, 1.64]	2.08	[2.02, 2.14]
Combined GE × Open Targets CT	cens.	I→II	1,466/1,626	54,687/65,988	1.09	[1.07, 1.11]	[1.06, 1.11]	1.89	[1.61, 2.23]
Combined GE × Open Targets CT	cens.	II→III	915/1,366	24,012/47,352	1.32	[1.27, 1.37]	[1.25, 1.39]	1.97	[1.76, 2.21]
Combined GE × Open Targets CT	cens.	III→Approved	444/826	5,760/19,824	1.85	[1.73, 1.98]	[1.68, 2.02]	2.84	[2.47, 3.26]
Combined GE × Open Targets CT	cens.	I→Approved	444/1,437	5,760/54,465	2.92	[2.69, 3.17]	[2.59, 3.28]	3.78	[3.37, 4.24]
Combined GE × Open Targets CT	uncens.	I→II	1,466/1,654	54,687/68,741	1.11	[1.09, 1.13]	[1.09, 1.14]	2.00	[1.72, 2.34]
Combined GE × Open Targets CT	uncens.	II→III	915/1,466	24,012/54,687	1.42	[1.36, 1.48]	[1.33, 1.51]	2.12	[1.91, 2.36]
Combined GE × Open Targets CT	uncens.	III→Approved	444/915	5,760/24,012	2.02	[1.89, 2.17]	[1.83, 2.23]	2.99	[2.62, 3.41]
Combined GE × Open Targets CT	uncens.	I→Approved	444/1,654	5,760/68,741	3.20	[2.95, 3.48]	[2.82, 3.61]	4.01	[3.59, 4.49]
Minikel et al. GE × Citeline CT	cens.	I→II	783/924	15,754/20,010	1.08	[1.05, 1.11]	[1.02, 1.13]	1.50	[1.25, 1.80]
Minikel et al. GE × Citeline CT	cens.	II→III	395/595	4,523/10,853	1.59	[1.50, 1.69]	[1.44, 1.76]	2.76	[2.32, 3.29]
Minikel et al. GE × Citeline CT	cens.	III→Approved	276/319	2,196/3,199	1.26	[1.20, 1.32]	[1.16, 1.36]	2.93	[2.11, 4.08]
Minikel et al. GE × Citeline CT	cens.	I→Approved	276/660	2,196/13,785	2.63	[2.38, 2.89]	[2.20, 3.11]	3.79	[3.23, 4.46]
Minikel et al. GE × Citeline CT	uncens.	I→II	783/1,031	15,754/23,144	1.12	[1.08, 1.16]	[1.05, 1.18]	1.48	[1.28, 1.71]
Minikel et al. GE × Citeline CT	uncens.	II→III	395/783	4,523/15,754	1.76	[1.63, 1.89]	[1.56, 1.97]	2.53	[2.19, 2.92]
Minikel et al. GE × Citeline CT	uncens.	III→Approved	276/395	2,196/4,523	1.44	[1.34, 1.55]	[1.27, 1.62]	2.46	[1.97, 3.07]
Minikel et al. GE × Citeline CT	uncens.	I→Approved	276/1,031	2,196/23,144	2.82	[2.53, 3.14]	[2.32, 3.38]	3.49	[3.02, 4.03]
Minikel et al. GE × Combined CT	cens.	I→II	17,615/18,816	1,934,605/2,117,862	1.02	[1.02, 1.03]	[1.02, 1.03]	1.39	[1.31, 1.47]
Minikel et al. GE × Combined CT	cens.	II→III	13,188/16,650	1,184,476/1,785,118	1.19	[1.18, 1.20]	[1.18, 1.21]	1.93	[1.86, 2.01]
Minikel et al. GE × Combined CT	cens.	III→Approved	9,208/12,507	622,420/1,075,940	1.27	[1.26, 1.29]	[1.25, 1.30]	2.03	[1.95, 2.12]
Minikel et al. GE × Combined CT	cens.	I→Approved	9,208/17,170	622,420/1,859,839	1.60	[1.58, 1.63]	[1.56, 1.65]	2.30	[2.23, 2.37]
Minikel et al. GE × Combined CT	uncens.	I→II	17,615/19,037	1,934,605/2,164,315	1.04	[1.03, 1.04]	[1.03, 1.04]	1.47	[1.39, 1.55]
Minikel et al. GE × Combined CT	uncens.	II→III	13,188/17,615	1,184,476/1,934,605	1.22	[1.21, 1.23]	[1.20, 1.24]	1.89	[1.82, 1.95]
Minikel et al. GE × Combined CT	uncens.	III→Approved	9,208/13,188	622,420/1,184,476	1.33	[1.31, 1.34]	[1.30, 1.36]	2.09	[2.01, 2.17]
Minikel et al. GE × Combined CT	uncens.	I→Approved	9,208/19,037	622,420/2,164,315	1.68	[1.66, 1.71]	[1.63, 1.73]	2.32	[2.26, 2.39]
Minikel et al. GE × Open Targets CT	cens.	I→II	819/939	55,334/66,675	1.05	[1.03, 1.08]	[1.02, 1.08]	1.40	[1.15, 1.70]
Minikel et al. GE × Open Targets CT	cens.	II→III	555/786	24,372/47,932	1.39	[1.33, 1.45]	[1.31, 1.47]	2.32	[1.99, 2.71]
Minikel et al. GE × Open Targets CT	cens.	III→Approved	294/527	5,910/20,123	1.90	[1.76, 2.06]	[1.71, 2.09]	3.03	[2.55, 3.61]
Minikel et al. GE × Open Targets CT	cens.	I→Approved	294/878	5,910/55,024	3.12	[2.83, 3.43]	[2.74, 3.54]	4.18	[3.63, 4.83]

Experiment	Cens.	Trans.	x_{yes}/n_{yes}	x_{no}/n_{no}	RS	RS 95% CI (Katz)	RS 95% CI (boot)	OR	OR 95% CI
Minikel et al. GE × Open Targets CT	uncens.	I→II	819/958	55,334/69,437	1.07	[1.04, 1.10]	[1.04, 1.11]	1.50	[1.25, 1.80]
Minikel et al. GE × Open Targets CT	uncens.	II→III	555/819	24,372/55,334	1.54	[1.47, 1.61]	[1.45, 1.63]	2.67	[2.30, 3.10]
Minikel et al. GE × Open Targets CT	uncens.	III→Approved	294/555	5,910/24,372	2.18	[2.01, 2.37]	[1.96, 2.42]	3.52	[2.97, 4.17]
Minikel et al. GE × Open Targets CT	uncens.	I→Approved	294/958	5,910/69,437	3.61	[3.27, 3.98]	[3.15, 4.08]	4.76	[4.14, 5.47]
Open Targets GE × Citeline CT	cens.	I→II	589/664	15,948/20,270	1.13	[1.10, 1.16]	[1.08, 1.17]	2.13	[1.67, 2.71]
Open Targets GE × Citeline CT	cens.	II→III	310/445	4,608/11,003	1.66	[1.56, 1.78]	[1.53, 1.81]	3.19	[2.59, 3.91]
Open Targets GE × Citeline CT	cens.	III→Approved	215/246	2,257/3,272	1.27	[1.20, 1.34]	[1.18, 1.36]	3.12	[2.12, 4.58]
Open Targets GE × Citeline CT	cens.	I→Approved	215/456	2,257/13,989	2.92	[2.63, 3.24]	[2.52, 3.37]	4.64	[3.84, 5.60]
Open Targets GE × Citeline CT	uncens.	I→II	589/736	15,948/23,439	1.18	[1.13, 1.22]	[1.12, 1.23]	1.88	[1.57, 2.26]
Open Targets GE × Citeline CT	uncens.	II→III	310/589	4,608/15,948	1.82	[1.68, 1.97]	[1.63, 2.02]	2.73	[2.32, 3.23]
Open Targets GE × Citeline CT	uncens.	III→Approved	215/310	2,257/4,608	1.42	[1.31, 1.53]	[1.26, 1.58]	2.36	[1.84, 3.02]
Open Targets GE × Citeline CT	uncens.	I→Approved	215/736	2,257/23,439	3.03	[2.69, 3.42]	[2.55, 3.55]	3.87	[3.28, 4.57]
Open Targets GE × Combined CT	cens.	I→II	11,920/12,505	1,940,300/2,124,173	1.04	[1.04, 1.05]	[1.04, 1.05]	1.93	[1.78, 2.10]
Open Targets GE × Combined CT	cens.	II→III	8,884/11,288	1,188,780/1,790,480	1.19	[1.17, 1.20]	[1.17, 1.20]	1.87	[1.79, 1.96]
Open Targets GE × Combined CT	cens.	III→Approved	5,879/8,389	625,749/1,080,058	1.21	[1.19, 1.23]	[1.18, 1.24]	1.70	[1.62, 1.78]
Open Targets GE × Combined CT	cens.	I→Approved	5,879/11,378	625,749/1,865,631	1.54	[1.51, 1.57]	[1.48, 1.60]	2.12	[2.04, 2.20]
Open Targets GE × Combined CT	uncens.	I→II	11,920/12,669	1,940,300/2,170,683	1.05	[1.05, 1.06]	[1.05, 1.06]	1.89	[1.75, 2.03]
Open Targets GE × Combined CT	uncens.	II→III	8,884/11,920	1,188,780/1,940,300	1.22	[1.20, 1.23]	[1.19, 1.24]	1.85	[1.78, 1.93]
Open Targets GE × Combined CT	uncens.	III→Approved	5,879/8,884	625,749/1,188,780	1.26	[1.24, 1.28]	[1.22, 1.29]	1.76	[1.68, 1.84]
Open Targets GE × Combined CT	uncens.	I→Approved	5,879/12,669	625,749/2,170,683	1.61	[1.58, 1.64]	[1.54, 1.68]	2.14	[2.06, 2.21]
Open Targets GE × Open Targets CT	cens.	I→II	1,140/1,269	55,013/66,345	1.08	[1.06, 1.10]	[1.05, 1.11]	1.82	[1.52, 2.19]
Open Targets GE × Open Targets CT	cens.	II→III	747/1,069	24,180/47,649	1.38	[1.32, 1.43]	[1.29, 1.46]	2.25	[1.97, 2.57]
Open Targets GE × Open Targets CT	cens.	III→Approved	378/675	5,826/19,975	1.92	[1.79, 2.06]	[1.74, 2.10]	3.09	[2.65, 3.61]
Open Targets GE × Open Targets CT	cens.	I→Approved	378/1,126	5,826/54,776	3.16	[2.90, 3.44]	[2.79, 3.56]	4.25	[3.74, 4.82]
Open Targets GE × Open Targets CT	uncens.	I→II	1,140/1,292	55,013/69,103	1.11	[1.09, 1.13]	[1.08, 1.14]	1.92	[1.62, 2.28]
Open Targets GE × Open Targets CT	uncens.	II→III	747/1,140	24,180/55,013	1.49	[1.43, 1.56]	[1.39, 1.59]	2.42	[2.14, 2.74]
Open Targets GE × Open Targets CT	uncens.	III→Approved	378/747	5,826/24,180	2.10	[1.95, 2.26]	[1.89, 2.32]	3.23	[2.79, 3.74]
Open Targets GE × Open Targets CT	uncens.	I→Approved	378/1,292	5,826/69,103	3.47	[3.18, 3.79]	[3.04, 3.94]	4.49	[3.97, 5.08]

Table S2: Clinical-trial source characterisation. Sources contribute to different legs, so they are tabulated separately. (**a**) The target–drug leg. The last column counts drugs carrying an `action_type` annotation in the parsed output, not a curated mechanism of action: ChEMBL's 0 means this extraction path (activities at pChEMBL ≥ 7) never joins the mechanism table, and STITCH's 9,509 is tagged by construction, having been filtered to activation and inhibition before the alias. (**b**) The drug–indication leg, by phase on the record, with the count of records whose latest phase is not recorded as concluded. (**c**) Target–indication *pairs* each arm contributes at each maximum phase: each arm re-aggregates that source on one leg with the other held at all sources, so `arm_pairs` is the whole arm table *before* the gene-universe restriction and *before* censoring, and does not reconcile against $n_{\text{yes}} + n_{\text{no}}$ by subtraction. Pair counts run far above the record counts in (b) — roughly sevenfold for TrialPanorama and nineteenfold for Open Targets — because the target–drug join maps one drug–indication record to many target–indication pairs. (**d**) Licences; per-source phase-matching rules are in `DATA_SOURCES.md`.

(a) Target–drug leg

Source	Edges	Genes	Drugs	Drugs w/ `action_type`
DGIdb	68,635	4,857	18,001	11,114
STITCH	34,829	2,994	9,509	9,509
ChEMBL	10,110	1,372	3,074	0
Open Targets	9,233	1,516	3,702	3,702
TrialPanorama	3,857	767	1,157	873

(b) Drug–indication leg

Source	Records	Drugs	EFO ids	Undet.	Ph. I	Ph. II	Ph. III	Ph. IV
TrialPanorama	270,725	34,013	3,570	51,873	52,219	107,131	60,728	50,647
Open Targets	36,845	3,683	2,611	7,438	7,973	15,133	9,154	4,585

(c) Target–indication pairs by maximum phase, per arm

Leg	Source	Arm pairs	maxϕ I	maxϕ II	maxϕ III	maxϕ IV	Ongoing
gene-drug	ChEMBL	235,214	37,300	106,714	45,358	45,842	38,139
gene-drug	DGIdb	1,543,745	170,749	542,688	405,983	424,325	224,138
gene-drug	STITCH	874,572	94,805	296,715	221,826	261,226	116,498
gene-drug	Open Targets	229,590	33,195	90,152	50,210	56,033	38,839
gene-drug	TrialPanorama	178,343	25,811	82,235	33,243	37,054	27,250
drug-indication	Open Targets	701,771	101,552	273,690	245,764	80,765	131,623
drug-indication	TrialPanorama	1,880,213	187,154	635,749	463,323	593,987	239,473
all	aggregate	2,236,441	238,399	774,268	578,681	645,093	314,272

(d) Licences

Domain	Source	License	Note
clinical_trials	Open Targets	CC0 1.0	known_drug is ChEMBL-derived; the Platform output is CC0
clinical_trials	TrialPanorama	Apache 2.0	HuggingFace dataset card only; no LICENSE file
clinical_trials	ChEMBL	CC BY-SA 3.0	share-alike propagates to any redistributed derivative
clinical_trials	STITCH	CC BY 4.0	covers actions, chemical_sources and protein_chemical_links; the *.detailed and *.transfer files are CC BY-NC-SA 4.0 and are not used
clinical_trials	DGIdb	code MIT; data per upstream source	interactions carry the licences of the sources DGIdb aggregates, some non-commercial; the parser drops interaction_source_db_name, recoverable from the raw `interactions.tsv` of the pinned 2024-Dec release
genetic_evidence	GWAS Catalog	EMBL-EBI terms; none named	EMBL-EBI 'places no additional restrictions on the use or redistribution of the data ... other than those provided by the original data owners'
genetic_evidence	ClinVar	US public domain; none named	'NCBI itself places no restrictions on the use or distribution of the data contained therein'; submitters may hold rights, attribution requested
genetic_evidence	Open Targets	CC0 1.0	association_by_datasource_direct, same 25.12 release
genetic_evidence	Genebass	none stated (unclear)	public bulk downloads of UK Biobank-derived summary statistics; the paper names no licence. Hail/GCS is an access burden, not a restriction

Table S3: Semantic-similarity matching-threshold sweep, phase I to approval. Tightening the match generally raises RS and reduces the supported count, so the 0.8 default is the conservative choice. Full per-transition rows are in the deposit.

Experiment	Threshold	$x_{\text{yes}}/n_{\text{yes}}$	RS	95% CI
Minikel et al. GE × Citeline CT	0.5	634/2,387	1.74	[1.61, 1.89]
Minikel et al. GE × Citeline CT	0.6	470/1,514	2.01	[1.84, 2.18]
Minikel et al. GE × Citeline CT	0.7	331/861	2.44	[2.22, 2.68]
Minikel et al. GE × Citeline CT	0.8	276/660	2.63	[2.38, 2.89]
Minikel et al. GE × Citeline CT	0.9	238/550	2.69	[2.43, 2.98]
Minikel et al. GE × Citeline CT	1.0	237/544	2.71	[2.44, 3.00]
Open Targets GE × Open Targets CT	0.5	1,243/7,467	1.63	[1.53, 1.72]
Open Targets GE × Open Targets CT	0.6	815/4,027	1.95	[1.82, 2.08]
Open Targets GE × Open Targets CT	0.7	551/2,026	2.59	[2.40, 2.79]
Open Targets GE × Open Targets CT	0.8	378/1,126	3.16	[2.90, 3.44]
Open Targets GE × Open Targets CT	0.9	273/750	3.38	[3.07, 3.73]
Open Targets GE × Open Targets CT	1.0	252/694	3.37	[3.04, 3.73]
Combined GE × Combined CT	0.5	71,470/193,350	1.11	[1.10, 1.12]
Combined GE × Combined CT	0.6	35,573/90,620	1.18	[1.17, 1.19]
Combined GE × Combined CT	0.7	16,144/36,777	1.31	[1.30, 1.33]
Combined GE × Combined CT	0.8	8,576/16,848	1.52	[1.50, 1.54]
Combined GE × Combined CT	0.9	5,246/9,570	1.63	[1.60, 1.66]
Combined GE × Combined CT	1.0	4,628/8,405	1.64	[1.61, 1.67]

Table S4: Oncology-stratified results, phase I to approval, for all nine cells of the 3×3 design. Oncology is MeSH tree C04 or the EFO neoplasm branch (Methods 2.2). Excluding oncology lowers the estimate in 30 of 36 cell-transitions. Full per-transition rows are in the deposit.

Experiment	Stratum	$x_{\text{yes}}/n_{\text{yes}}$	$x_{\text{no}}/n_{\text{no}}$	RS	95% CI
Minikel et al. GE × Citeline CT	with oncology	276/660	2,196/13,785	2.63	[2.38, 2.89]
Minikel et al. GE × Citeline CT	without oncology	262/623	1,309/7,667	2.46	[2.22, 2.73]
Combined GE × Citeline CT	with oncology	247/581	2,225/13,864	2.65	[2.39, 2.93]
Combined GE × Citeline CT	without oncology	222/502	1,349/7,788	2.55	[2.29, 2.85]
Open Targets GE × Citeline CT	with oncology	215/456	2,257/13,989	2.92	[2.63, 3.24]
Open Targets GE × Citeline CT	without oncology	193/398	1,378/7,892	2.78	[2.48, 3.11]
Minikel et al. GE × Open Targets CT	with oncology	294/878	5,910/55,024	3.12	[2.83, 3.43]
Minikel et al. GE × Open Targets CT	without oncology	275/821	4,208/29,072	2.31	[2.09, 2.56]
Combined GE × Open Targets CT	with oncology	444/1,437	5,760/54,465	2.92	[2.69, 3.17]
Combined GE × Open Targets CT	without oncology	382/1,144	4,101/28,749	2.34	[2.15, 2.55]
Open Targets GE × Open Targets CT	with oncology	378/1,126	5,826/54,776	3.16	[2.90, 3.44]
Open Targets GE × Open Targets CT	without oncology	323/909	4,160/28,984	2.48	[2.26, 2.71]
Minikel et al. GE × Combined CT	with oncology	9,208/17,170	622,420/1,859,839	1.60	[1.58, 1.63]
Minikel et al. GE × Combined CT	without oncology	8,898/16,328	531,390/1,209,609	1.24	[1.22, 1.26]
Combined GE × Combined CT	with oncology	8,576/16,848	623,052/1,860,161	1.52	[1.50, 1.54]
Combined GE × Combined CT	without oncology	7,631/13,255	532,657/1,212,682	1.31	[1.29, 1.33]
Open Targets GE × Combined CT	with oncology	5,879/11,378	625,749/1,865,631	1.54	[1.51, 1.57]
Open Targets GE × Combined CT	without oncology	5,157/8,887	535,131/1,217,050	1.32	[1.30, 1.34]

Table S5: Convergence check against Tsepilov et al. (2026), run under their cohort definition (ChEMBL-derived pairs, curated mechanism-of-action targets, phase IV vs. I–III, oncology excluded, exact indication matching, no censoring). Their published GWAS cell is 742 supported / 242 approved of 37,377, against 4,322 of 36,635 unsupported; they report RS 2.76 and OR 3.62. The 95% intervals for that cell quoted here, [2.48, 3.08] and [3.09, 4.23], are computed from those counts with the Katz and Woolf estimators used throughout. Our three evidence definitions bracket their value.

Evidence definition	Trans.	$x_{\text{yes}}/n_{\text{yes}}$	RS	95% CI	OR
GWAS credible sets, propagated ≥ 0.5	I→II	649/740	1.05	[1.03, 1.08]	1.44
Open Targets GE, direct ≥ 0.5	I→II	577/632	1.10	[1.07, 1.12]	2.12
Combined GE	I→II	719/786	1.10	[1.08, 1.12]	2.17
Open Targets GE, direct ≥ 0.5	II→III	410/577	1.34	[1.27, 1.41]	2.17
Combined GE	II→III	499/719	1.31	[1.25, 1.38]	2.01
GWAS credible sets, propagated ≥ 0.5	II→III	451/649	1.31	[1.24, 1.38]	2.02
Open Targets GE, direct ≥ 0.5	III→Approved	215/410	2.00	[1.82, 2.20]	3.10
GWAS credible sets, propagated ≥ 0.5	III→Approved	221/451	1.87	[1.69, 2.06]	2.70
Combined GE	III→Approved	256/499	1.96	[1.80, 2.15]	2.98
GWAS credible sets, propagated ≥ 0.5	I→Approved	221/740	2.57	[2.30, 2.89]	3.24
Open Targets GE, direct ≥ 0.5	I→Approved	215/632	2.94	[2.63, 3.29]	3.94
Combined GE	I→Approved	256/786	2.83	[2.55, 3.14]	3.71

Table S6: Germline-only versus with-somatic genetic evidence, phase I to approval. Germline-only is the headline; including `eva_somatic` and `intogen` moves the estimate as shown. Full per-transition rows are in the deposit.

Evidence scope	Clinical trials	$x_{\text{yes}}/n_{\text{yes}}$	RS	95% CI
Open Targets, germline	Citeline CT	215/456	2.92	[2.63, 3.24]
Open Targets, with somatic	Citeline CT	229/485	2.94	[2.66, 3.25]
Combined, germline (headline)	Citeline CT	247/581	2.65	[2.39, 2.93]
Combined, + OT somatic	Citeline CT	260/606	2.68	[2.43, 2.96]
Open Targets, germline	Open Targets CT	378/1,126	3.16	[2.90, 3.44]
Open Targets, with somatic	Open Targets CT	393/1,235	2.99	[2.75, 3.26]
Combined, germline (headline)	Open Targets CT	444/1,437	2.92	[2.69, 3.17]
Combined, + OT somatic	Open Targets CT	458/1,538	2.82	[2.60, 3.05]
Open Targets, germline	Combined CT	5,879/11,378	1.54	[1.51, 1.57]
Open Targets, with somatic	Combined CT	6,138/12,551	1.46	[1.43, 1.48]
Combined, germline (headline)	Combined CT	8,576/16,848	1.52	[1.50, 1.54]
Combined, + OT somatic	Combined CT	8,819/17,957	1.47	[1.44, 1.49]

Table S7: Clinical-trial source comparison by leg, phase I to approval, on combined genetic evidence. The indication leg decides the estimate; the target–drug leg does not. Base rate is the unsupported progression rate. Full per-transition rows are in the deposit.

Leg	Source	$x_{\text{yes}}/n_{\text{yes}}$	RS	95% CI	OR	Base rate
gene_drug	ChEMBL	713/1,845	1.67	[1.58, 1.77]	2.09	0.23
gene_drug	DGIdb	6,685/13,308	1.57	[1.54, 1.59]	2.14	0.32
gene_drug	STITCH	3,494/6,923	1.47	[1.44, 1.51]	1.95	0.34
gene_drug	Open Targets	1,151/2,545	1.55	[1.49, 1.62]	2.01	0.29
gene_drug	TrialPanorama	646/1,560	1.70	[1.60, 1.80]	2.19	0.24
gene_drug	All	8,576/16,848	1.52	[1.50, 1.54]	2.06	0.33
drug_indication	Open Targets	2,608/8,973	2.09	[2.02, 2.16]	2.53	0.14
drug_indication	TrialPanorama	7,575/13,521	1.55	[1.53, 1.57]	2.25	0.36
drug_indication	All	8,576/16,848	1.52	[1.50, 1.54]	2.06	0.33

Table S8: Censoring conventions on the Minikel et al. GE × Citeline CT arm. Entry-gate censoring is defined at phase I entry, so on the single-transition rows it coincides with per-transition censoring at I→II and with no censoring at II→III and III→Approved; on the I→Approved composite all three differ, and are compared directly.

Convention	Trans.	$x_{\text{yes}}/n_{\text{yes}}$	$x_{\text{no}}/n_{\text{no}}$	RS	95% CI
no censoring (Tsepilov et al.)	I→II	783/1,031	15,754/23,144	1.12	[1.08, 1.16]
entry gate only (Minikel et al.)	I→II	783/924	15,754/20,010	1.08	[1.05, 1.11]
per transition (this work)	I→II	783/924	15,754/20,010	1.08	[1.05, 1.11]
entry gate only (Minikel et al.)	II→III	395/783	4,523/15,754	1.76	[1.63, 1.89]
per transition (this work)	II→III	395/595	4,523/10,853	1.59	[1.50, 1.69]
no censoring (Tsepilov et al.)	II→III	395/783	4,523/15,754	1.76	[1.63, 1.89]
entry gate only (Minikel et al.)	III→Approved	276/395	2,196/4,523	1.44	[1.34, 1.55]
no censoring (Tsepilov et al.)	III→Approved	276/395	2,196/4,523	1.44	[1.34, 1.55]
per transition (this work)	III→Approved	276/319	2,196/3,199	1.26	[1.20, 1.32]
no censoring (Tsepilov et al.)	I→Approved	276/1,031	2,196/23,144	2.82	[2.53, 3.14]
entry gate only (Minikel et al.)	I→Approved	276/924	2,196/20,010	2.72	[2.45, 3.03]
per transition (this work)	I→Approved	276/660	2,196/13,785	2.63	[2.38, 2.89]

Table S9: Dataset coverage. Pairs, genes and EFO ids per source, raw and within the protein-coding gene universe.

Domain	Source	Pairs	Genes	EFO ids	Pairs (univ.)	Genes (univ.)	EFO (univ.)
genetic evidence	Minikel (Citeline)	107,365	12,523	2,265	104,294	11,721	2,263
clinical trials	Minikel (Citeline)	43,962	2,462	1,174	43,498	2,393	1,172
genetic evidence	Combined	552,778	30,721	23,792	427,844	17,963	23,517
genetic evidence	GWAS Catalog	395,722	28,919	12,653	274,663	16,877	12,304
genetic evidence	ClinVar	20,890	3,919	4,364	17,285	3,137	4,361
genetic evidence	Open Targets	227,775	14,796	16,702	227,584	14,714	16,695
genetic evidence	Genebass	3,281	1,483	154	3,155	1,442	154
clinical trials	Open Targets (known_drug)	70,537	1,515	2,611	70,395	1,509	2,608
clinical trials	Combined	2,236,441	5,336	4,568	2,183,352	5,082	4,567

Table S10: Clustered-bootstrap sensitivity on the headline arm: resampling genes versus indications (10,000 replicates, seed 0). Indication clustering widens intervals further, and every interval still excludes unity.

Cluster	Trans.	RS	95% CI (Katz)	95% CI (boot)
efo_id	I→II	1.04	[1.03, 1.04]	[1.03, 1.04]
efo_id	II→III	1.18	[1.17, 1.19]	[1.15, 1.20]
efo_id	III→Approved	1.21	[1.20, 1.23]	[1.17, 1.25]
efo_id	I→Approved	1.52	[1.50, 1.54]	[1.44, 1.59]
gene	I→II	1.04	[1.03, 1.04]	[1.03, 1.04]
gene	II→III	1.18	[1.17, 1.19]	[1.16, 1.19]
gene	III→Approved	1.21	[1.20, 1.23]	[1.19, 1.24]
gene	I→Approved	1.52	[1.50, 1.54]	[1.47, 1.57]

Table S11: Enrichment by individual genetic-evidence source. Every source × comparator combination at each phase transition, under per-transition censoring, with relative success (RS), its Katz 95% CI, the odds ratio and its Woolf 95% CI. Genebass rests on the smallest counts; at phase I to approval its interval overlaps every other source in the Citeline comparator, all but ClinVar in the Combined comparator, and only ClinVar in the Open Targets comparator.

Comparator	Source	Trans.	$x_{\text{yes}}/n_{\text{yes}}$	$x_{\text{no}}/n_{\text{no}}$	RS	RS 95% CI	OR	OR 95% CI
Citeline CT	ClinVar	I→II	154/161	16,383/20,773	1.21	[1.17, 1.25]	5.90	[2.76, 12.58]
Citeline CT	ClinVar	II→III	102/120	4,816/11,328	2.00	[1.85, 2.16]	7.66	[4.64, 12.66]
Citeline CT	ClinVar	III→Approved	77/80	2,395/3,438	1.38	[1.32, 1.45]	11.18	[3.52, 35.50]
Citeline CT	ClinVar	I→Approved	77/105	2,395/14,340	4.39	[3.89, 4.96]	13.72	[8.88, 21.18]
Citeline CT	Open Targets	I→II	589/664	15,948/20,270	1.13	[1.10, 1.16]	2.13	[1.67, 2.71]
Citeline CT	Open Targets	II→III	310/445	4,608/11,003	1.66	[1.56, 1.78]	3.19	[2.59, 3.91]
Citeline CT	Open Targets	III→Approved	215/246	2,257/3,272	1.27	[1.20, 1.34]	3.12	[2.12, 4.58]
Citeline CT	Open Targets	I→Approved	215/456	2,257/13,989	2.92	[2.63, 3.24]	4.64	[3.84, 5.60]
Citeline CT	GWAS Catalog	I→II	330/411	16,207/20,523	1.02	[0.97, 1.07]	1.08	[0.85, 1.39]
Citeline CT	GWAS Catalog	II→III	155/261	4,763/11,187	1.39	[1.26, 1.55]	1.97	[1.54, 2.53]
Citeline CT	GWAS Catalog	III→Approved	99/117	2,373/3,401	1.21	[1.12, 1.31]	2.38	[1.43, 3.96]
Citeline CT	GWAS Catalog	I→Approved	99/304	2,373/14,141	1.94	[1.64, 2.29]	2.39	[1.88, 3.06]
Citeline CT	Genebass	I→II	15/18	16,522/20,916	1.05	[0.86, 1.30]	1.33	[0.38, 4.60]
Citeline CT	Genebass	II→III	8/11	4,910/11,437	1.69	[1.18, 2.43]	3.54	[0.94, 13.37]
Citeline CT	Genebass	III→Approved	6/7	2,466/3,511	1.22	[0.90, 1.65]	2.54	[0.31, 21.15]
Citeline CT	Genebass	I→Approved	6/13	2,466/14,432	2.70	[1.50, 4.86]	4.16	[1.40, 12.39]
Open Targets CT	ClinVar	I→II	213/232	55,940/67,382	1.11	[1.06, 1.15]	2.29	[1.43, 3.67]
Open Targets CT	ClinVar	II→III	136/201	24,791/48,517	1.32	[1.20, 1.46]	2.00	[1.49, 2.69]
Open Targets CT	ClinVar	III→Approved	77/116	6,127/20,534	2.22	[1.95, 2.54]	4.64	[3.15, 6.83]
Open Targets CT	ClinVar	I→Approved	77/200	6,127/55,702	3.50	[2.93, 4.18]	5.07	[3.81, 6.74]
Open Targets CT	Open Targets	I→II	1,140/1,269	55,013/66,345	1.08	[1.06, 1.10]	1.82	[1.52, 2.19]
Open Targets CT	Open Targets	II→III	747/1,069	24,180/47,649	1.38	[1.32, 1.43]	2.25	[1.97, 2.57]
Open Targets CT	Open Targets	III→Approved	378/675	5,826/19,975	1.92	[1.79, 2.06]	3.09	[2.65, 3.61]
Open Targets CT	Open Targets	I→Approved	378/1,126	5,826/54,776	3.16	[2.90, 3.44]	4.25	[3.74, 4.82]
Open Targets CT	GWAS Catalog	I→II	741/819	55,412/66,795	1.09	[1.07, 1.12]	1.95	[1.54, 2.47]
Open Targets CT	GWAS Catalog	II→III	452/682	24,475/48,036	1.30	[1.23, 1.37]	1.89	[1.61, 2.22]
Open Targets CT	GWAS Catalog	III→Approved	214/419	5,990/20,231	1.73	[1.57, 1.90]	2.48	[2.04, 3.01]
Open Targets CT	GWAS Catalog	I→Approved	214/727	5,990/55,175	2.71	[2.42, 3.04]	3.43	[2.91, 4.03]
Open Targets CT	Genebass	I→II	19/21	56,134/67,593	1.09	[0.95, 1.25]	1.94	[0.45, 8.33]
Open Targets CT	Genebass	II→III	16/19	24,911/48,699	1.65	[1.35, 2.00]	5.09	[1.48, 17.48]
Open Targets CT	Genebass	III→Approved	12/16	6,192/20,634	2.50	[1.88, 3.32]	7.00	[2.26, 21.70]
Open Targets CT	Genebass	I→Approved	12/21	6,192/55,881	5.16	[3.56, 7.47]	10.70	[4.51, 25.40]
Combined CT	ClinVar	I→II	1,522/1,617	1,950,698/2,135,061	1.03	[1.02, 1.04]	1.51	[1.23, 1.86]
Combined CT	ClinVar	II→III	1,027/1,384	1,196,637/1,800,384	1.12	[1.08, 1.15]	1.45	[1.29, 1.64]
Combined CT	ClinVar	III→Approved	546/927	631,082/1,087,520	1.01	[0.96, 1.07]	1.04	[0.91, 1.18]
Combined CT	ClinVar	I→Approved	546/1,379	631,082/1,875,630	1.18	[1.10, 1.26]	1.29	[1.16, 1.44]
Combined CT	Open Targets	I→II	11,920/12,505	1,940,300/2,124,173	1.04	[1.04, 1.05]	1.93	[1.78, 2.10]
Combined CT	Open Targets	II→III	8,884/11,288	1,188,780/1,790,480	1.19	[1.17, 1.20]	1.87	[1.79, 1.96]
Combined CT	Open Targets	III→Approved	5,879/8,389	625,749/1,080,058	1.21	[1.19, 1.23]	1.70	[1.62, 1.78]
Combined CT	Open Targets	I→Approved	5,879/11,378	625,749/1,865,631	1.54	[1.51, 1.57]	2.12	[2.04, 2.20]
Combined CT	GWAS Catalog	I→II	9,553/10,109	1,942,667/2,126,569	1.03	[1.03, 1.04]	1.63	[1.49, 1.77]
Combined CT	GWAS Catalog	II→III	7,353/9,161	1,190,311/1,792,607	1.21	[1.20, 1.22]	2.06	[1.95, 2.17]

Comparator	Source	Trans.	$x_{\text{yes}}/n_{\text{yes}}$	$x_{\text{no}}/n_{\text{no}}$	RS	RS 95% CI	OR	OR 95% CI
Combined CT	GWAS Catalog	III→Approved	5,147/6,967	626,481/1,081,480	1.28	[1.26, 1.29]	2.05	[1.95, 2.17]
Combined CT	GWAS Catalog	I→Approved	5,147/9,331	626,481/1,867,678	1.64	[1.61, 1.67]	2.44	[2.34, 2.54]
Combined CT	Genebass	I→II	156/169	1,952,064/2,136,509	1.01	[0.97, 1.06]	1.13	[0.64, 2.00]
Combined CT	Genebass	II→III	120/150	1,197,544/1,801,618	1.20	[1.11, 1.30]	2.02	[1.35, 3.01]
Combined CT	Genebass	III→Approved	84/112	631,544/1,088,335	1.29	[1.16, 1.44]	2.17	[1.41, 3.33]
Combined CT	Genebass	I→Approved	84/155	631,544/1,876,854	1.61	[1.39, 1.86]	2.33	[1.70, 3.20]
